# Impact of severity of bronchial asthma on oral health in children

**DOI:** 10.3389/froh.2025.1594568

**Published:** 2025-09-03

**Authors:** Murad Alrashdi, Abdullah Alyahya

**Affiliations:** 1 Department of Orthodontic and Peadiatric Dentistry, College of Dentistry, Qassim University, Burayadh, Saudi Arabia; 2 College of Medicine, Hail University, Hail, Saudi Arabia

**Keywords:** dental caries, gingival index, pediatric dentistry, asthma, oral health

## Abstract

**Background:**

The relationship between asthma and oral health has garnered increasing attention due to overlapping risk factors, such as altered immune responses and behavioral changes.

**Objective:**

To assess and compare the oral health status of children with bronchial asthma to their healthy siblings and unrelated healthy controls in the Qassim region, KSA.

**Materials and methods:**

180 participants were included in the present study and were divided into 3 groups as follows: Group A: 60 children with confirmed diagnosis of long-standing bronchial asthma, group B: 60 healthy subjects who served as a negative control group, group C: 60 healthy siblings of asthmatic children served as a Sibling control group (participants had the same dietary patterns and socioeconomic standards as participants of group A). Dental caries were assessed using the Decayed, Missing, and Filled Teeth (dmft/DMFT) index, which is a standardized and widely used measure of Decayed, Missing, and Filled Teeth in dental epidemiology. Gingival health was evaluated via the Gingival Index (GI).

**Results:**

*Post hoc* analysis was applied to evaluate the differences between the 3 studied groups regarding DMF score and gingival index and all these differences were found to be highly significant. Patients with bronchial asthma (Group A) were further subdivided into 3 subgroups according to severity of asthma (25 patients had mild asthma, 23 patients had moderate asthma, and 12 patients had severe asthma) based on GINA guidelines. Strikingly, all the differences between these 3 subgroups regarding demographic data, DMF score and gingival index were all insignificant. Further evaluation of each subgroup was done by applying *post hoc* analysis and each of the 3 subgroups was found to be significantly different than the positive and negative control groups regarding DMF score and gingival index.

**Conclusion:**

Bronchial asthma has direct and indirect effect (related to drug therapy) on oral health. Dealing with some modifiable cofactors could be helpful in improving oral health in children with asthma.

## Introduction

Bronchial asthma is one of the most common chronic respiratory diseases, affecting an estimated 14% of children worldwide and contributing significantly to global morbidity and healthcare costs (1–3).

Asthma is characterized by airway inflammation, bronchial hyperresponsiveness, and reversible airway obstruction. These pathophysiological changes are often triggered by environmental allergens, infections, and or genetic predispositions. In children, asthma poses unique challenges due to its chronic nature and the impact it has on their growth, daily activities, and overall quality of life (1, 4, 5).

The relationship between asthma and oral health has garnered increasing attention due to overlapping risk factors, such as altered immune responses and behavioral changes (6, 7).

Chronic oral health issues, including dental caries and periodontal diseases, have been observed with higher prevalence in asthmatic populations. This association may stem from the frequent use of inhaled corticosteroids, reduced salivary flow, and mouth breathing, which can create a conducive environment for oral pathogens (2, 4, 8).

Inhaled corticosteroids, a cornerstone of asthma management, are associated with significant changes in the oral microbiome. This results in an increased risk of dental caries, particularly in children who are less likely to adhere to rigorous oral hygiene practices (4, 9).

Saliva plays a crucial role in oral health, with defensive mechanisms that can be impaired in patients with asthma due to drug therapy, alimentary habits and/or lifestyles. Saliva is important for maintaining a neutral pH in the oral cavity. In addition, salivary flow allows mechanical cleansing of food debris. Oral clearance is greatly related to the rate of salivary secretion. These defensive mechanisms were found to be impaired in patient with asthma (beta 2 agonists have a negative impact on salivary production rate and some inhalers have a low pH (10).

Despite these observations, existing research has not adequately explored the correlation between asthma severity and oral health outcomes. Children with mild asthma may experience less pronounced oral health challenges compared to those with severe symptoms (4, 11).

Previous studies investigating oral health in children with asthmatic had multiple limitations, such as small sample sizes and a lack of bronchial asthma severity stratification (1, 4, 12). Additionally, few studies have comprehensively assessed the impact of behavioral factors, such as oral hygiene practices and dietary habits, in children with asthma.

The main aim of this study was to address these gaps by systematically comparing the oral health status of children with asthma to their healthy peers while accounting for the severity of asthma.

Given the prevalence of asthma and its potential to exacerbate oral health issues, a deeper understanding of these interactions is essential for developing integrated healthcare strategies.

This study is particularly considering the growing evidence linking chronic oral health conditions to systemic inflammation and overall quality of life, emphasizing the need for a multidisciplinary approach to pediatric healthcare (2, 6, 13).

## Aim of the study

To assess and compare the oral health status of children with bronchial asthma to their healthy siblings and unrelated healthy controls in the Qassim region, KSA. The inclusion of healthy siblings of asthmatic children as a comparison group is a novel approach intended to control for shared dietary patterns and socioeconomic background, helping isolate the specific impact of asthma.

## Patients and methods

This study is a cross-sectional observational study conducted at a single point in time. Participants were recruited using stratified random sampling from pediatric clinics and hospitals in the Qassim region, and categorized into three groups based on asthma diagnosis and family relationship.

Participants were recruited from pediatric outpatient clinics and government hospitals across the Qassim region between January and April 2025. Asthmatic participants (Group A) were identified based on a confirmed diagnosis of bronchial asthma by a pulmonologist using the GINA 2024 guidelines. Healthy control participants (Group B) were recruited from general pediatric visits and had no history of asthma or other chronic conditions. Sibling controls (Group C) were identified through Group A families, ensuring one healthy sibling per asthmatic child. All participants were screened for eligibility according to inclusion and exclusion criteria prior to enrollment. Informed consent was obtained from parents or legal guardians before participation.

The study involving 180 children, aged 4–12 years, residing in the Qassim region, KSA. The required sample size was calculated using Cochran's formula for comparison between three independent groups. Assuming a medium effect size (Cohen's *f* = 0.25), a confidence level of 95%, and power of 80%, a minimum of 159 participants was required. To account for potential dropouts and incomplete data, the final sample size was increased to 180, with 60 participants in each group.

Participants were randomly recruited from pediatric clinics and hospitals within the Qassim region of KSA and were divided into 3 groups as follows:

- Group A: 60 children with confirmed diagnosis of long-standing bronchial asthma, stratified by asthma severity [25 patients (41.7%) had mild asthma, 23 patients (38.3%) had moderate asthma, and 12 patients (20%) had severe asthma] based on GINA (Global Initiative for Asthma) guidelines (1).
- Group B: 60 healthy subjects served as a negative control group.
- Group C: 60 healthy siblings of asthmatic children served as a Sibling control group (participants had the same dietary patterns and socioeconomic standards as participants of group A).

While Group C (healthy siblings of asthmatic children) was included specifically to control for shared dietary habits and socioeconomic status, Group B (unrelated healthy controls) was not matched for these factors. Participants in Group B were randomly selected from pediatric clinics across different neighborhoods within the Qassim region. Socioeconomic and dietary variability in this group was acknowledged and addressed as a potential limitation of the study.

All participants were assessed by a dentist who was blind to all clinical, laboratory and radiological findings. The examiner was a board-certified pediatric dentist with over five years of clinical experience. Prior to data collection, the examiner underwent a calibration session using 10 pilot cases to ensure consistency in DMFT and gingival index scoring. Intra-examiner reliability was assessed and confirmed with a kappa score of >0.85, indicating strong agreement.

Dental caries were assessed using the dmft index for primary teeth and the DMFT index for permanent teeth, following WHO criteria (14). Each child was examined for the presence of decayed (d/D), missing (m/M), and filled (f/F) teeth, and the total score was recorded. If a child had a mixed dentition, both indices were used and reported separately for analysis consistency. Children with complete absence of index teeth or those with full edentulism were excluded from the study.

Gingival health was assessed using the Gingival Index (GI) developed by Löe and Silness (1963), evaluating four surfaces per tooth (mesial, distal, buccal, lingual) and scoring inflammation from 0 to 3. The average GI score was calculated per participant. Examinations were performed under adequate lighting with sterile mouth mirrors and explorers.

The Gingival Index (GI), developed by Löe and Silness in 1963 (15), is a widely used and established clinical tool to assess gingival inflammation. It does not require a separate questionnaire form but instead involves a clinical examination form where scores are recorded.

Gingival Index evaluates: Color, Consistency, Bleeding on probing. Each gingival unit (mesial, distal, buccal, and lingual) of a tooth is scored on a scale from 0 to 3:

- 0: Normal gingiva (no inflammation).
- 1: Mild inflammation (slight color change, no bleeding).
- 2: Moderate inflammation (redness, swelling, and bleeding on probing).
- 3: Severe inflammation (marked redness, swelling, ulceration, spontaneous bleeding).

The scores are averaged for the teeth or regions examined to give a GI score.

A structured questionnaire was used to collect information on oral hygiene practices, dietary patterns, and medication use. The questionnaire was adapted from previously validated tools used in pediatric dental and asthma-related studies. It was reviewed by two pediatric dentistry faculty members and piloted on a sample of 10 parents for clarity and relevance. The final version was administered in Arabic and completed by the parents or primary caregivers of the children, with assistance from trained dental interns when needed. Data quality was ensured by real-time checking for completeness and consistency by the supervising examiner during the interview.

All participants were evaluated by a pulmonologist who was blind to the Oro-dental status of the participants. GINA Asthma Severity and Control Classification (1) was implemented to classify asthma severity (mild, moderate, or severe) as follows (Table 1):

**Table 1:** GINA asthma severity and control classification.

Parameters	Frequency/Severity	Notes
Daytime symptoms	<2 days/week; >2 days/week	
Night-time waking	None; <2×/month; >2×/month	
Reliever use	<2×/week; >2×/week	
Activity limitation	None; Mild; Moderate	
Pulmonary function (FEV1)	Normal; 60%–80%; <60%	Based on spirometry

Participants in Group A were receiving treatment for bronchial asthma according to the NICE 2021 guidelines (16). Treatment protocols were stratified based on severity as follows:

Mild asthma (*n* = 25): Managed primarily with short-acting beta-agonists (SABA) on an as-needed basis, with occasional use of low-dose inhaled corticosteroids (ICS). Moderate asthma (*n* = 23): Treated with daily low-to-moderate dose ICS combined with long-acting beta-agonists (LABA). Severe asthma (*n* = 12): Required high-dose ICS-LABA therapy, with several patients receiving intermittent courses of oral corticosteroids due to exacerbations.

Subgrouping within Group A was based on the Global Initiative for Asthma (GINA) 2024 classification, which considers symptom frequency, medication use, and pulmonary function. The rationale for subgrouping was to explore whether asthma severity correlates with differences in oral health status (DMFT score and gingival index). This aligns with the study's objective to determine not only the presence of an association between asthma and oral health but also whether this association varies by clinical severity. Subgroup analysis allows for a more granular understanding of the potential dose-response relationship between asthma burden and oral health outcomes.

The average age at first asthma diagnosis was 3.8 years (range: 2–6 years), and the mean duration of treatment prior to participation was 3.5 years. Children with other systemic conditions affecting immunity or salivary function were excluded from the study.

Inclusion criteria: Children aged 4–12 years residing in the Qassim region. For Group A, participants had to have a confirmed diagnosis of bronchial asthma for more than one year. Group B participants had to be healthy with no chronic illness. Group C included healthy siblings of Group A participants, matched by family and living environment.

Exclusion criteria were as follows: children with chronic inflammatory conditions (e.g., rheumatic fever, cystic fibrosis), recent dental treatment (defined as any restorative, periodontal, or surgical dental procedure within the past 3 months), recent antibiotic use, or evidence of malnutrition (defined as BMI-for-age below the 5th percentile according to WHO growth reference standards).

Randomization and sibling structure: Stratified random sampling was used to select participants within each group to reduce selection bias. For Group C, only one healthy sibling was selected per asthmatic child to avoid repeated familial influence on the dataset. Participants were randomly selected from a master list generated from clinic databases and family registries using a random number generator.

This study was approved by the Hail University Institutional Review Board (IRB Approval No. H-2025-606). Written informed consent was obtained from the parents or legal guardians of all participating children prior to enrollment. All procedures were conducted in accordance with the ethical standards of the Declaration of Helsinki.

### Statistical analysis

Statistical analysis was performed using SPSS version 23 (IBM Corp., Chicago, IL). Quantitative variables were expressed as mean ± standard deviation (SD) or median with interquartile range (IQR) based on data distribution. Qualitative variables were expressed as frequencies and percentages.

The Shapiro–Wilk test was used to assess the normality of continuous variables. For comparisons between the three main groups (Group A: asthmatic children, Group B: healthy controls, Group C: healthy siblings), one-way ANOVA was used for normally distributed continuous variables (e.g., Gingival Index), and the Kruskal–Wallis test was used for non-normally distributed variables (e.g., DMFT scores). *Post hoc* comparisons were performed using Fisher's Least Significant Difference (LSD) test. Chi-square tests were used for categorical comparisons (e.g., gender distribution across groups).

To compare the asthma subgroups (mild, moderate, severe) within Group A, one-way ANOVA or Kruskal–Wallis tests were used based on variable distribution, followed by *post hoc* analysis was performed using Fisher's Least Significant Difference (LSD) test to identify specific pairwise differences between groups. Correlation analysis was conducted using Spearman's rank correlation to assess associations between asthma severity and oral health indices. A *p*-value of ≤0.05 was considered statistically significant, and *p* < 0.01 was considered highly significant.

## Results

180 participants were included in the present study and were divided into 3 groups as follows:

Group A: 60 children with confirmed diagnosis of long-standing bronchial asthma, group B: 60 healthy subjects who served as a negative control group, group C: 60 healthy siblings of asthmatic children served as a Sibling control group (participants had the same dietary patterns and socioeconomic standards as participants of group A).

As for the demographic data, the current study revealed insignificant differences between the 3 studied groups as regard age and gender (*P*-value = 0.600 and 0.818 respectively) (Table 2). This similarity in age and gender distribution across groups reduces the likelihood that demographic factors influenced the observed oral health differences.

**Table 2:** Comparison between negative control, positive control and asthmatic groups regarding age and gender distribution.

Parameter		Negative control	Positive control	Asthmatic	Test value	*P*-value	Sig.
		No. = 60	No. = 60	No. = 60			
Age	Mean ± SD	7.58 ± 2.55	8.05 ± 2.5	7.78 ± 2.55	0.513**	0.600	NS
	Range	4–12	4–12	4–12			
Gender	Male	27 (45%)	27 (45%)	30 (50%)	0.402*	0.818	NS
	Female	33 (55%)	33 (55%)	30 (50%)			

NS, non-significant; Sig., significance, No, number.

* Chi-square test.

** One Way ANOVA test. Insignificant differences were found between the 3 studied groups as regard age and gender.

Initial comparison between the three groups was conducted using one-way ANOVA for normally distributed variables (Gingival Index) and the Kruskal–Wallis test for non-normally distributed variables (DMFT score). Following significant results, *post hoc* analysis was performed using Fisher's Least Significant Difference (LSD) test to identify pairwise differences.

**Figure 1:**
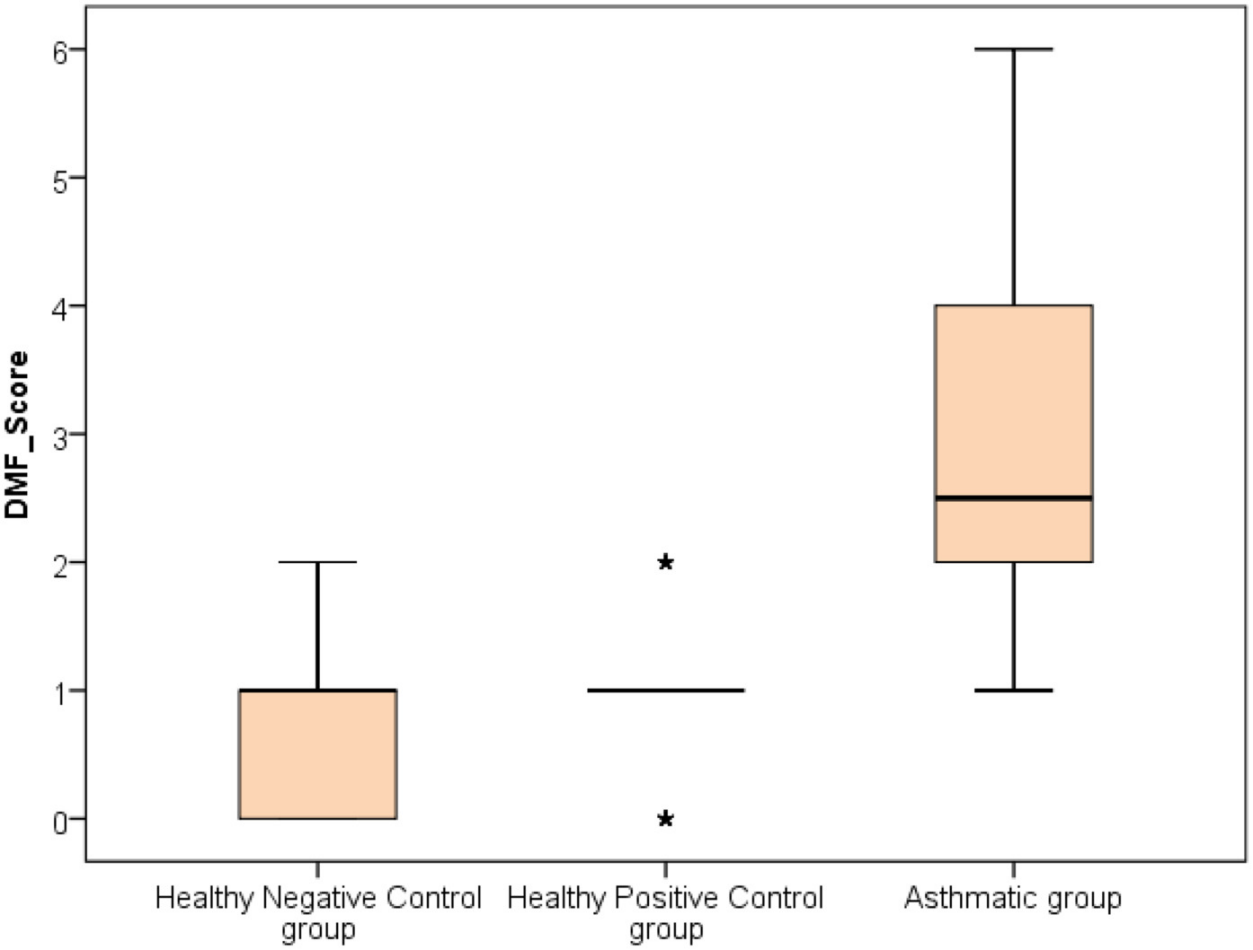
Comparison between negative control, positive control and asthmatic groups regarding DMF score.

**Figure 2:**
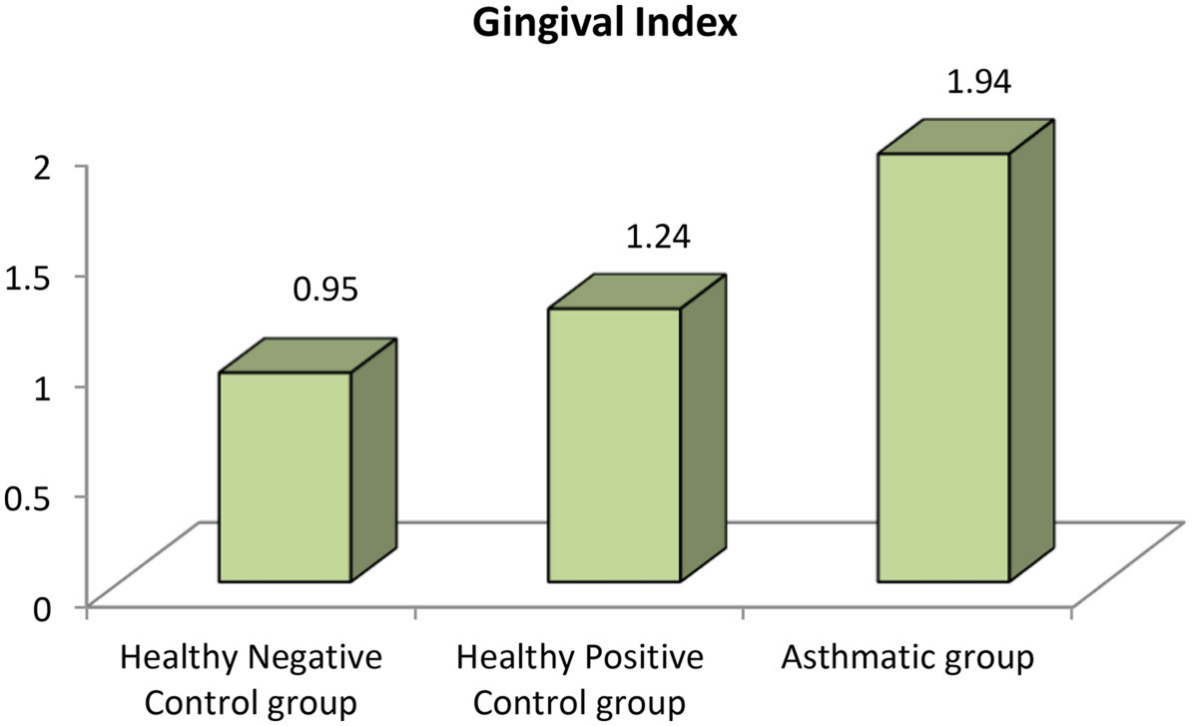
Comparison between negative control, positive control and asthmatic groups regarding gingival index.

The analysis revealed that both asthmatic children and their healthy siblings had significantly higher DMFT scores and gingival index values compared to healthy controls (*p* < 0.01). Clinically, this underscores that children with asthma — and even their siblings — may be more vulnerable to caries and gingival inflammation, likely due to shared environmental or behavioral factors. These findings highlight the importance of targeted oral health interventions in families affected by asthma (negative control group vs. Sibling control group, negative control group vs. asthmatic group, Sibling control group vs. asthmatic group) (*P*-value <0.01 for all) (Table 3) (Figures 1, 2).

**Table 3:** Comparison between the 3 studied groups regarding DMF score and gingival index.

Parameter		Negative control group	Positive control group	Asthmatic group	Test value	*P*-value	Sig.
		No. = 60	No. = 60	No. = 60			
DMF score	Median (IQR)	1 (0–1)	1 (1–1)	2.5 (2–4)	84.185**	0.000	HS
	Range	0–2	0–2	1–6			
Gingival index	Mean ± SD	0.95 ± 0.1	1.24 ± 0.1	1.94 ± 0.42	235.913*	0.000	HS
	Range	0.8–1.1	1.1–1.4	1.4–2.8			
*Post hoc* analysis by LSD and multi-comparison between groups							
Parameters		P1		P2		P3	
DMF score		0.006		0.000		0.000	
Gingival index		0.000		0.000		0.000	

HS, highly-significant; Sig., significance; No., number; P1, negative control group vs. sibling control group; P2, negative control group vs. asthmatic group; P3, sibling control group vs. asthmatic group.

Highly significant differences were found between the 3 studied groups regarding DMF score and gingival index.

* One Way ANOVA test.

** Kruskal–Wallis test.

*** Chi-square test.

Clinically, these findings indicate that children with asthma exhibit nearly double the gingival inflammation levels (mean GI ∼1.9) compared to healthy peers (mean GI ∼0.95). Similarly, the higher DMFT scores suggest a markedly increased risk for untreated decay or restorative needs in asthmatic children.

GINA Asthma Severity and Control Classification (1) was implemented to classify group A according to asthma severity into 3 subgroups: mild, moderate, or severe. 25 patients (41.7%) had mild asthma, 23 patients (38.3%) had moderate asthma, and 12 patients (20%) had severe asthma Strikingly, all the differences between these 3 subgroups regarding demographic data, DMF score and gingival index were all insignificant (*P*-value >0.05) (Table 4).

**Table 4:** Relation of asthma severity to demographic data, DMF score and gingival index.

Parameter		Asthma Severity			Test value	*P*-value	Sig.
		Mild	Moderate	Severe			
		No. = 25	No. = 23	No. = 12			
Age	Mean ± SD	7 ± 2.55	8.39 ± 2.33	8.25 ± 2.7	2.120*	0.129	NS
	Range	4–12	4–12	4–12			
Gender	Male	14 (56%)	9 (39.1%)	7 (58.3%)	1.780***	0.411	NS
	Female	11 (44%)	14 (60.9%)	5 (41.7%)			
DMF score	Median (IQR)	2 (2–4)	3 (2–5)	2 (1.5–3.5)	0.808**	0.668	NS
	Range	1–6	1–6	1–6			
Gingival index	Mean ± SD	1.92 ± 0.4	1.97 ± 0.42	1.95 ± 0.49	0.083*	0.921	NS
	Range	1.4–2.6	1.4–2.6	1.4–2.8			

NS, non-significant; Sig., significance; No., number.

Asthma severity had insignificant relation to demographic data, DMF score or gingival index.

* One Way ANOVA test.

** Kruskal–Wallis test.

*** Chi-square test.

Each subgroup was further compared to positive and negative control groups regarding age and gender and all the differences were statistically insignificant (*P*-value >0.05) (Tables 5–7). By ensuring demographic comparability between groups, the study minimized the potential confounding effect of age and gender on oral health outcomes.

**Table 5:** Comparison between negative controls, positive controls and mild asthmatic groups regarding all studied parameters.

Parameter		Negative Control group	Sibling control group	Mild asthmatic group	Test value	*P*-value	Sig.
		No. = 60		No. = 25			
Age	Mean ± SD	7.58 ± 2.55	8.05 ± 2.5	7 ± 2.55	1.587*	0.208	NS
	Range	4–12	4–12	4–12			
Gender	Male	27 (45%)	27 (45%)	14 (56%)	1.005***	0.605	NS
	Female	33 (55%)	33 (55%)	11 (44%)			
DMF score	Median (IQR)	1 (0–1)	1 (1–1)	2 (2–4)	48.175**	0.000	HS
	Range	0–2	0–2	1–6			
Gingival index	Mean ± SD	0.95 ± 0.1	1.24 ± 0.1	1.92 ± 0.4	227.370*	0.000	HS
	Range	0.8–1.1	1.1–1.4	1.4–2.6			
*Post hoc* analysis							
Parameter		P1		P2		P3	
DMF score		0.006		0.000		0.000	
Gingival index		0.000		0.000		0.000	

NS, non-significant; HS, highly significant; No., number; Sig., significance; P1, negative control vs. positive control; P2, negative control vs. mild asthma; P3, positive control vs. mild asthma.

Highly significant differences were found between patients with mild asthma and both control groups regarding DMF Score and Gingival Index.

* One Way ANOVA test.

** Kruskal–Wallis test.

*** Chi-square test.

**Table 6:** Comparison between negative control, positive control and moderate asthmatic groups regarding all studied parameters.

Parameter		Negative Control group	Sibling control group	Moderate asthmatic group	Test value	*P*-value	Sig.
		No. = 60		No. = 25			
Age	Mean ± SD	7.58 ± 2.55	8.05 ± 2.5	8.39 ± 2.33	1.035*	0.358	NS
	Range	4–12	4–12	4–12			
Gender	Male	27 (45%)	27 (45%)	9 (39.1%)	0.270***	0.874	NS
	Female	33 (55%)	33 (55%)	14 (60.9%)			
DMF score	Median (IQR)	1 (0–1)	1 (1–1)	3 (2–5)	48.556**	0.000	HS
	Range	0–2	0–2	1–6			
Gingival index	Mean ± SD	0.95 ± 0.1	1.24 ± 0.1	1.97 ± 0.42	234.348*	0.000	HS
	Range	0.8–1.1	1.1–1.4	1.4–2.6			
*Post hoc* analysis							
Parameter		P1		P2		P3	
DMF score		0.006		0.000		0.000	
Gingival index		0.000		0.000		0.000	

NS, non-significant; HS, highly significant; Sig., significant; No., number; P1, negative control vs. positive control; P2, negative control vs. moderate asthma; P3, positive control vs. moderate asthma.

Highly significant differences were found between patients with moderate asthma and both control groups regarding DMF Score and Gingival Index.

* One way ANOVA test.

** Kruskal–Wallis test.

*** Chi-square test.

**Table 7:** Comparison between negative control, positive control and severe asthmatic groups regarding all studied parameters.

Parameter		Healthy negative control group	Healthy Sibling control group	Severe asthmatic	Test value	*P*-value	Sig.
		No. = 60		No. = 25			
Age	Mean ± SD	7.58 ± 2.55	8.05 ± 2.5	8.25 ± 2.7	0.664*	0.517	NS
	Range	4–12	4–12	4–12			
Gender	Male	27 (45%)	27 (45%)	7 (58.3%)	0.780***	0.677	NS
	Female	33 (55%)	33 (55%)	5 (41.7%)			
DMF score	Median (IQR)	1 (0–1)	1 (1–1)	2 (1.5–3.5)	27.141**	0.000	HS
	Range	0–2	0–2	1–6			
Gingival index	Mean ± SD	0.95 ± 0.1	1.24 ± 0.1	1.95 ± 0.49	171.127*	0.000	HS
	Range	0.8–1.1	1.1–1.4	1.4–2.8			
*Post hoc* analysis							
Parameter		P1		P2		P3	
DMF score		0.006		0.000		0.000	
Gingival index		0.000		0.000		0.000	

NS, non-significant; HS, highly significant; Sig., significant; No., number; P1, negative control vs. positive control; P2, negative control vs. severe asthma; P3, positive control vs. severe asthma.

Highly significant differences were found between patients with severe asthma and both control groups regarding DMF Score and Gingival Index.

* One way ANOVA test.

** Kruskal–Wallis test.

*** Chi-square test.

Although differences between mild, moderate, and severe asthma subgroups were statistically insignificant, all asthma subgroups consistently showed worse oral health outcomes compared to sibling and healthy controls. This pattern reinforces that asthma itself — regardless of severity — may predispose children to poor oral health.

**Figure 3:**
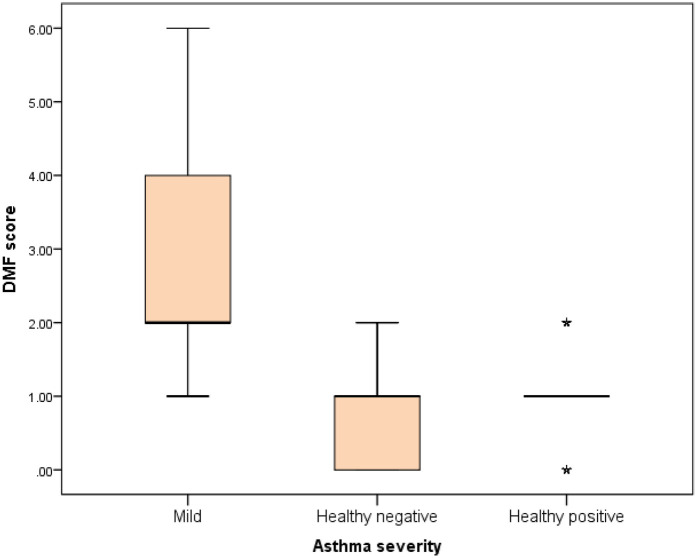
Comparison between negative control, positive control and mild asthmatic groups regarding DMF score.

**Figure 4:**
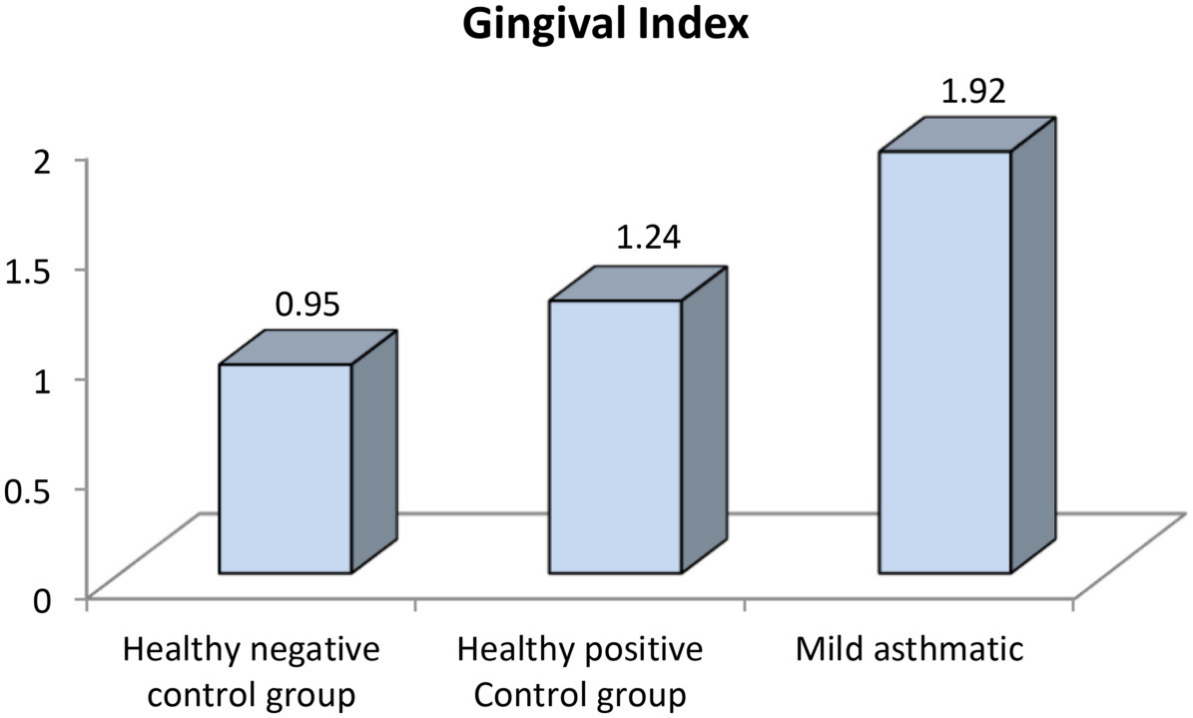
Comparison between negative control, positive control and mild asthmatic groups regarding gingival index.

**Figure 5:**
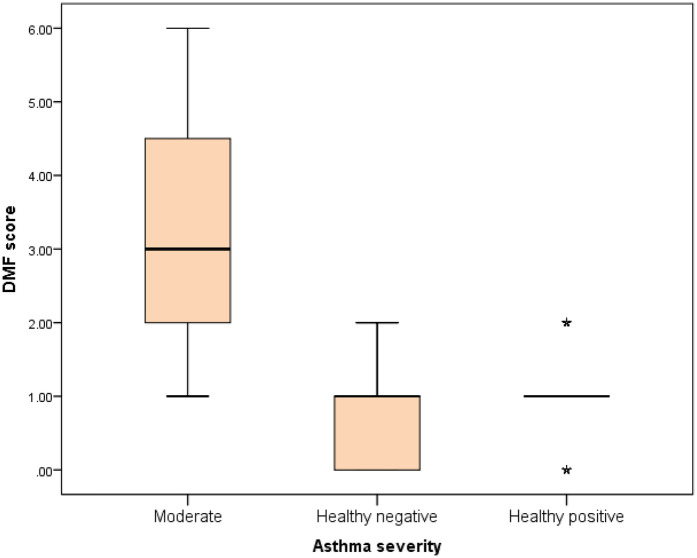
Comparison between negative control, positive control and moderate asthmatic groups regarding DMF score.

**Figure 6:**
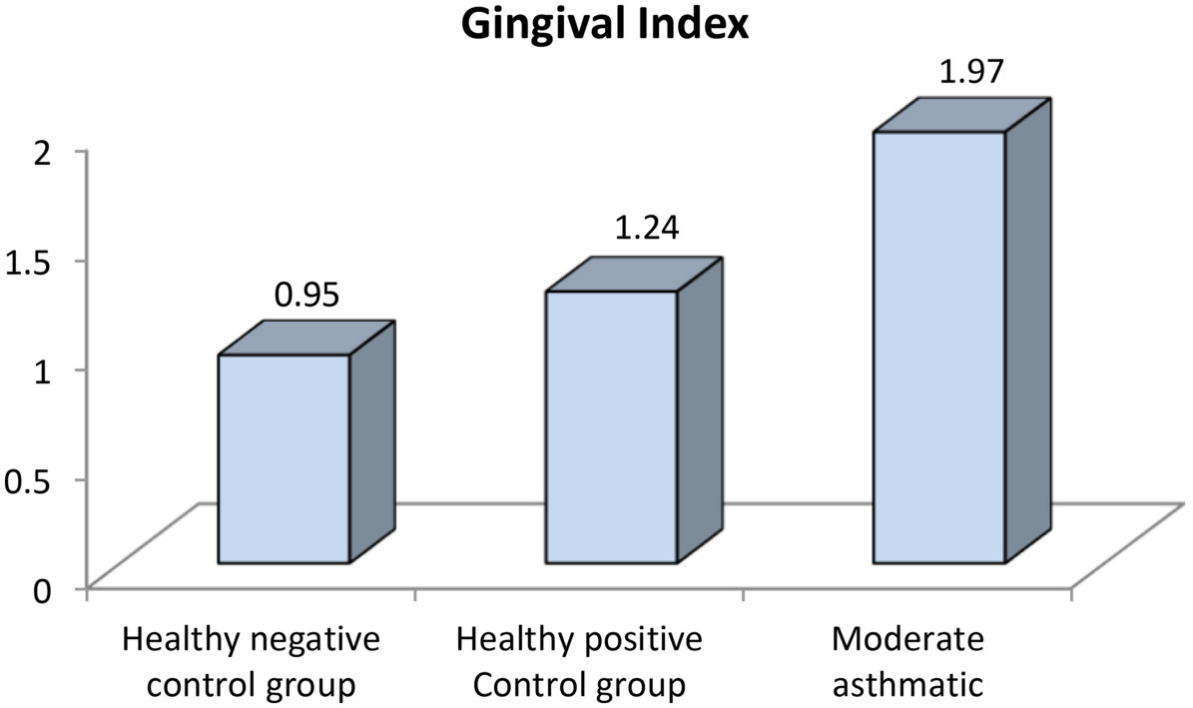
Comparison between negative controls, positive control and moderate asthmatic groups regarding gingival index.

**Figure 7:**
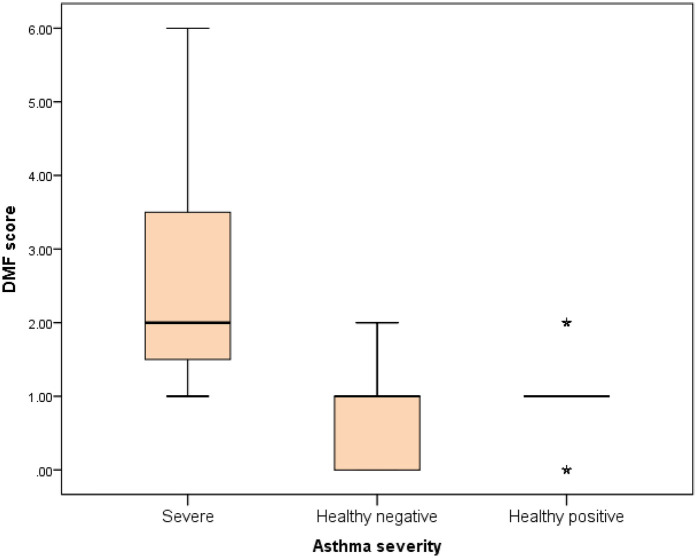
Comparison between negative control, positive control and severe asthmatic groups regarding DMF score.

**Figure 8:**
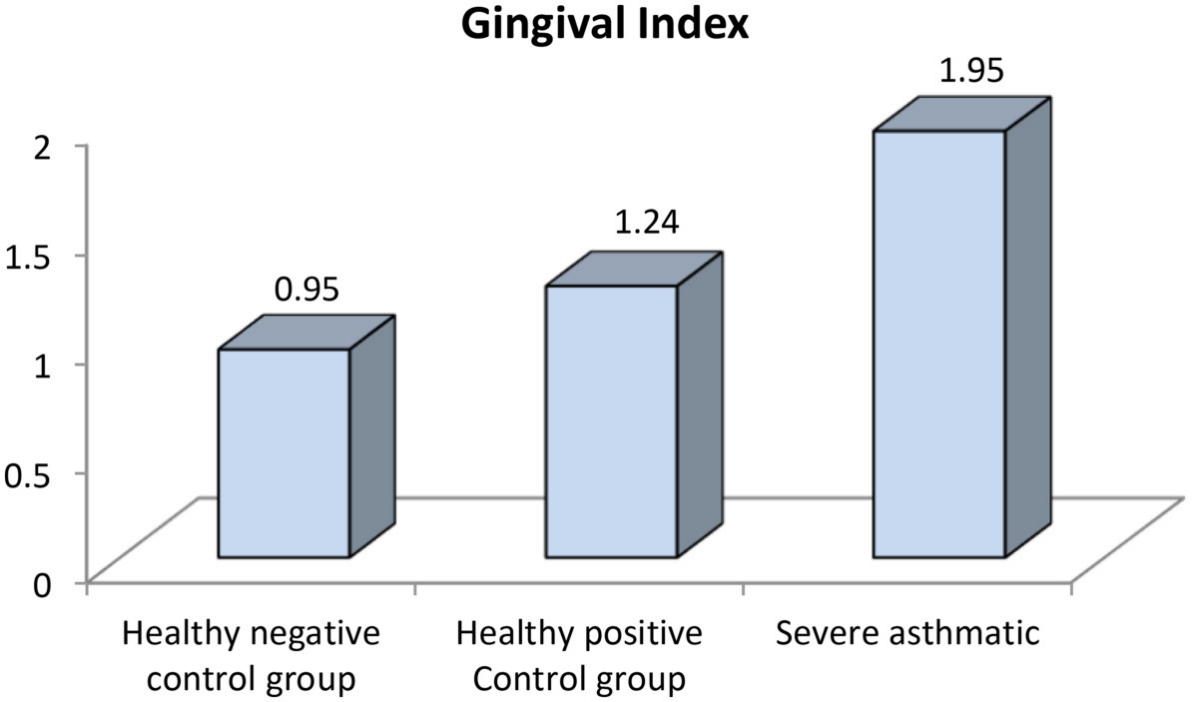
Comparison between negative control, positive control and severe asthmatic groups regarding gingival index.

Further evaluation of each subgroup was done by applying *post hoc* Analysis and each of the 3 subgroups was significantly different than the positive and negative control groups regarding DMF score and gingival index (*P*-value <0.01 for each subgroup) (Tables 5–7) (Figures 3–8).

## Discussion

Bronchial asthma is a chronic inflammatory disease characterized by increased airway responsiveness to different environmental triggers and by symptoms such as wheezing, chest tightness and dyspnea. The diagnosis is based on clinical evaluation and demonstration of reversible airway obstruction, either spontaneously or after treatment. Treatment is tailored according to the severity of the disease. This includes short acting bronchodilators, anti-inflammatory drugs such as corticosteroid inhalers (oral corticosteroids used only in selected severe cases), long-acting bronchodilators, and leukotriene antagonists. Only severe forms of asthma should receive biologic agents (17). These drugs can have significant impact on oral health through different mechanisms, increasing the risk of caries, dental erosion, tooth loss, periodontal disease and oral-pharyngeal candidiasis. Moreover, patients with bronchial asthmatic, may show alterations in salivary components such as reduction in total proteins, amylase, hexosamine, salivary peroxidase, lysozymes and secretory IgA level (18).

Asthma and its pharmacological management, particularly with inhaled corticosteroids and beta-agonists, can lead to xerostomia, altered salivary pH, and microbial imbalance, all of which increase the risk of caries and gingival inflammation. Moreover, neglected oral hygiene and chronic inflammation in the oral cavity have been proposed as potential contributors to systemic inflammatory burden, which could theoretically worsen asthma control. While this bidirectional relationship requires further investigation, it emphasizes the need for integrated dental-medical care in asthmatic children.

The current study was conducted to assess and compare the oral health status of children with bronchial asthma to their healthy siblings and unrelated healthy controls in the Qassim region. 180 participants were included: 60 children with confirmed diagnosis of long-standing bronchial asthma, 60 healthy subjects served as a negative control group and 60 healthy siblings of children with asthma served as a Sibling control group (these participants had the same dietary patterns and socioeconomic standards as children with asthma). Insignificant differences were found between the three studied groups as regard age and gender and these results helped to eliminate the effect of other confounders (such as age and gender) on the oral health.

Our findings align with studies from other regions such as Europe and North America, which have also reported higher caries and gingival disease prevalence in children with asthma (e.g., Sagheri et al.; Arafa et al.), (6, 19). This suggests that the oral health challenges faced by asthmatic children may be a broader, possibly global concern, influenced by common mechanisms such as medication use and behavioral patterns.

While high sugar consumption is common among all children, some studies suggest that asthmatic children may have increased exposure to cariogenic diets. A study by Sagheri et al. reported significantly higher sugar intake frequency in asthmatic children compared to healthy peers, possibly linked to behavioral factors and medication-related oral dryness (6, 19). However, this association remains context-dependent and warrants further investigation. Furthermore, dental erosion may be associated with the use of beta 2 agonists, but cofactors like lifestyle, associated drugs, or GERD can increase the risk (20).

According to the NICE 2021 guidelines, asthma management in children typically involves stepwise use of inhaled corticosteroids (ICS), short-acting beta-agonists (SABA), long-acting beta-agonists (LABA), and, in more severe cases, oral corticosteroids or leukotriene receptor antagonists (LTRAs). These medications, particularly ICS, have been associated with oral side effects such as dry mouth, reduced salivary flow, and increased susceptibility to oral candidiasis. These factors may collectively elevate caries risk and contribute to periodontal inflammation if oral hygiene measures are inadequate.

Confirming the role of lifestyle and other cofactors such as genetic pattern and socioeconomic status, the current study revealed a significant difference between siblings of patients with asthma and healthy subjects regarding DMF Score and Gingival Index. As some of these cofactors are modifiable, Interventions such as neutral sodium fluoride mouth rinses after using inhalers (18), using spacer devices, and treating GERD, could be helpful in treating and or preventing dental erosion in children with asthma (21).

Numerous studies revealed that bronchial asthma itself (irrespective to drug therapy, lifestyle or socioeconomic status) can be considered a major risk for increased gingival inflammation (19). The results of the current study added great evidence to these reports as patients with asthma were significantly different than their siblings and normal subjects regarding DMF Score and Gingival Index.

Several factors can explain this finding: 1- alteration of immune response with an increased concentration of IgE and a reduction of secretory IgA, 2- mouth breathing (18), 3- the alteration of salivary composition, or the reduction of salivary flow (19). All these factors can promote the interaction between bacterial and immunological elements. An increased level of IgE in the gingiva and higher levels of calcium and phosphorous with higher prevalence of calculus in the saliva, can also be involved in poor periodontal health (21).

Although our study did not measure dental erosion directly, previous studies have linked asthma and its medications—particularly inhaled corticosteroids—to increased risk of dental erosion and caries. These findings support the biological plausibility of our observed association between asthma and poorer oral health, as measured by higher DMFT scores and gingival inflammation. A British study conducted in 2000 aimed to assess the level of dental erosion in a sample of 418 participants, 15.8% of them were asthmatics. It concluded that the level of dental erosion was higher in asthmatic children than in control group (22). Another study did not demonstrate a significant difference in dental erosion between asthmatic children and healthy controls (23). These data highlight the importance of other cofactors besides drug therapy in the development of dental erosion. A cross-sectional study, conducted in 2017 (18-year-olds), detected erosion in 42.3% of participants. The statistical analysis revealed that the prevalence of erosion in the anterior region was related to gastroesophageal reflux, eating disorders, and asthma (24).

Another cross-sectional study involving 400 children (6–14 years old), evaluated the relationship between dental erosion and multiple etiological factors. The patients' risk level of developing dental erosion was obtained from the BEWE index. A significant relationship between the intake of carbonated beverages, isotonic drinks and fruit juices and a higher BEWE was detected (20).

The relationship between severity of bronchial asthma and oral health remains controversial. While some studies confirm a direct relation between asthma severity and oral health, others failed to prove such a relation. A study published in 2017 showed that gingival pathology (considering the Gingival Index) was more prevalent in children with asthma than in healthy subjects and that it increases with the severity of asthma (19).

A retrospective study involving 19,206 patients with asthma, found that the risk of developing periodontal disease increased dramatically for patients with more severe form of asthma (multiple emergency department visits and/or hospital admission). An association between inhaled corticosteroids and increased risk of periodontal disease was also detected (25). However, this study had several limitations including lack of information about the severity of the disease, the dose of inhaled corticosteroids, and the short follow up period of patients.

Interestingly, no statistically significant differences were found between mild, moderate, and severe asthma subgroups with respect to DMFT scores or gingival index. This suggests that the presence of asthma itself — rather than its clinical severity — may be the dominant factor influencing oral health outcomes in children. However, it is important to interpret this result cautiously, as the small sample size in the severe asthma subgroup (*n* = 12) may have limited the statistical power to detect subtle differences. Future studies with larger and more evenly distributed severity groups are warranted to validate these findings.

In conclusion, this study confirms that children with bronchial asthma have significantly poorer oral health—demonstrated by higher DMFT scores and gingival index—compared to both healthy unrelated children and their own healthy siblings. The inclusion of a sibling control group is a unique strength, helping to control for shared environmental and socioeconomic factors and highlighting the likely influence of asthma itself and its treatment on oral health outcomes. Interestingly, no significant differences were observed between asthma severity subgroups, suggesting that the presence of asthma may be a more critical factor than its severity. These findings underscore the need for early oral health monitoring and preventive care in asthmatic children. Future longitudinal studies are warranted to further explore the mechanisms involved and the long-term effects of asthma management on oral health.

## Strengths and limitations

This study has several strengths that are worth mentioning. First, inclusion of a Sibling control group which was composed of healthy siblings of asthmatic children who had the same dietary patterns and socioeconomic standards as their siblings. Second, all types of asthma severity were included (with different pattern of drug therapy). Third, both DMF score and gingival index were evaluated to validate the results.

On the other hand, we acknowledge few limitations of this study. First is the limited heterogeneity of the three studied groups. Indeed, all participants originated from the Qassim region of KSA. Future multicenter studies with a larger, more diverse sample could clarify this issue. Second, oral health was not directly correlated with other important items such as renal function tests, vitamin D level serum calcium, magnesium and phosphate. However, results of the current study emphasize the importance of conduction of further research taking into consideration both the issue. Finally, the number of participants in each of the three sub-group of asthma was relatively small to obtain significant results.

Additionally, while the inclusion of sibling controls (Group C) helped control for shared socioeconomic and dietary factors, the unrelated healthy control group (Group B) was not matched for these variables. This may have introduced confounding and should be considered when interpreting group differences.

A further limitation is the lack of baseline oral health history for participants. Because the study was cross-sectional, it could not assess whether oral health changes were progressive or linked temporally to asthma onset or treatment. Longitudinal studies are needed to explore causality and disease progression over time.

## Data Availability

The raw data supporting the conclusions of this article will be made available by the authors, without undue reservation.
